# Fast and optimal branch-and-bound planner for the grid-based coverage path planning problem based on an admissible heuristic function

**DOI:** 10.3389/frobt.2022.1076897

**Published:** 2023-01-27

**Authors:** Jaël Champagne Gareau, Éric Beaudry, Vladimir Makarenkov

**Affiliations:** GDAC-LIA, Computer Science Department, Université du Québec à Montréal, Montréal, QC, Canada

**Keywords:** coverage path planning (CPP), robotics, iterative deepening depth-first search, branch-and-bound, heuristic search, optimal solution, pruning, intelligent decision making

## Abstract

This paper introduces an optimal algorithm for solving the discrete grid-based coverage path planning (CPP) problem. This problem consists in finding a path that covers a given region completely. First, we propose a CPP-solving baseline algorithm based on the iterative deepening depth-first search (ID-DFS) approach. Then, we introduce two branch-and-bound strategies (Loop detection and an Admissible heuristic function) to improve the results of our baseline algorithm. We evaluate the performance of our planner using six types of benchmark grids considered in this study: Coast-like, Random links, Random walk, Simple-shapes, Labyrinth and Wide-Labyrinth grids. We are first to consider these types of grids in the context of CPP. All of them find their practical applications in real-world CPP problems from a variety of fields. The obtained results suggest that the proposed branch-and-bound algorithm solves the problem optimally (i.e., the exact solution is found in each case) orders of magnitude faster than an exhaustive search CPP planner. To the best of our knowledge, no general CPP-solving exact algorithms, apart from an exhaustive search planner, have been proposed in the literature.

## 1 Introduction

The field of automated planning (sometimes called *AI planning*) focuses on finding a sequence of actions that allows an intelligent agent (for example, a robot) to reach a goal state (for example, a specific position in the environment) from a given initial state (Ghallab et al., 2016). An example of a real-world application of automated planning is the problem of finding an optimal path for an electric vehicle (Champagne Gareau et al., 2021a). Another problem studied in automated planning is the complete Coverage Path-Planning (CPP) problem, where the objective is to find an optimal or quasi-optimal path that covers every area in the region (we call such a path a *complete coverage path* of the region). This problem has many practical applications, such as:

a) robotic vacuum-cleaning (Viet et al., 2013; Yakoubi and Laskri, 2016; Edwards and Sörme, 2018; Liu et al., 2018);
b) underwater autonomous vehicles (AUVs) (Zhu et al., 2019; Han et al., 2020; Yordanova and Gips, 2020);
c) 3d printing using fused deposition modeling (Lechowicz et al., 2016; Afzal et al., 2019; Gupta, 2021);
d) window washer robots (Farsi et al., 1994; Dr.; John Dhanaseely and Srinivasan, 2021);
e) disinfection of regions (Conroy et al., 2021; Nasirian et al., 2021; Vazquez-Carmona et al., 2022);
f) minesweeping (Healey, 2001; Williams, 2010; Ðakulovic and Petrovic, 2012);
g) agriculture and farming (Oksanen and Visala, 2009; Jin, 2010; Santos et al., 2020);
h) surveillance drones (Ahmadzadeh et al., 2008; Modares et al., 2017; Vasquez-Gomez et al., 2018);
i) search and rescue aerial drones (Hayat et al., 2020; Ai et al., 2021; Cho et al., 2021).

**FIGURE 1:**
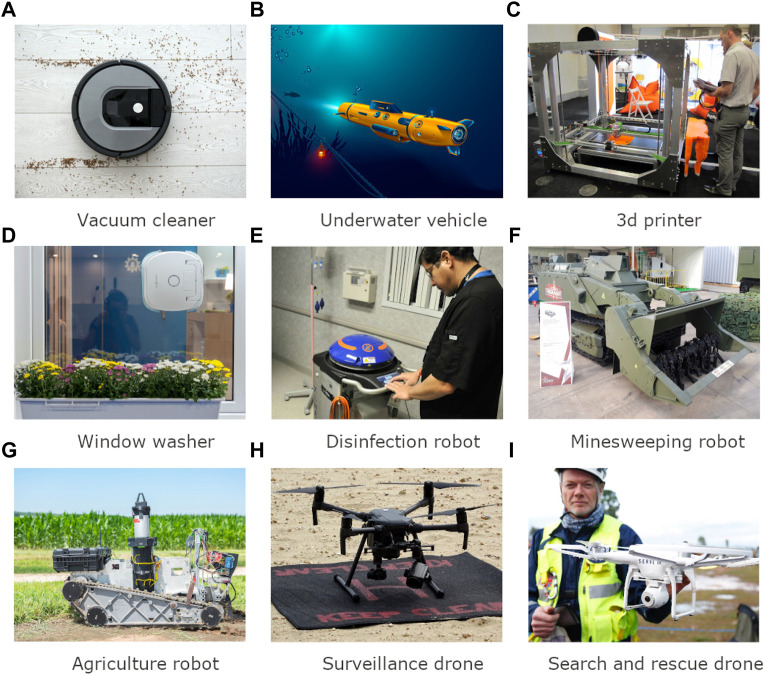
Visual representation of CPP practical applications (all photos were taken from the public domain).

Figure 1 gives a visual representation of these applications. All of them rely on efficient CPP algorithms to accomplish their task (Choset, 2001; Galceran and Carreras, 2013; Khan et al., 2017).

Many variants of the CPP problem exist. The algorithm to be used depends a lot on the variant under study. The working environment can be either discrete (e.g., grid-based, graph-based, etc.) or continuous, 2D or 3D, known *a priori* (i.e., off-line algorithms), or discovered while doing the coverage (i.e., on-line algorithms). Moreover, the coverage can be done by a single agent, or by the cooperation of multiple agents. Some variants also restrict the type of allowed movements or add different kinds of sensors to the agent (e.g., proximity sensor, GPS, gyroscopic sensor, etc.). Some variants even consider positional uncertainties and energetic constraints of the agent. In this paper, we focus on the classical variant consisting of a single agent in a 2D discrete grid-based environment with no specific constraints or uncertainties.

The objective of our study is to present an optimal CPP planner that runs orders of magnitude faster than a naive search algorithm through the state-space. Our main research contributions are as follows. We propose:

1) A novel branch-and-bound optimal planner to the grid CPP problem;
2) An informative, admissible, efficient heuristic to the grid CPP problem;
3) Realistic environments for discrete grid-based CPP benchmarking.

This article is an extended version of our conference paper presented at the IDEAL2021 meeting (Champagne Gareau et al., 2021b). We extend our previous work by:

• greatly expanding the Introduction and Related Work sections to present various applications of CPP and to better show how our algorithm (including the two proposed improvements) compares to the existing approaches;
• improving the proposed heuristic function;
• improving the empirical evaluation, including additionnal experiments, new plots and two new type of benchmark grids;
• expanding the analysis of our results, by explaining more deeply the causes of the observed improvements and what these results mean in practice.

Below, we present an overview of existing CPP solving approaches and their practical applications in the field of robotics.

Grid-based methods decompose the environment that we want to explore into a collection of uniform grid cells. A grid-based representation was first proposed by Moravec and Elfes (1985). A classical algorithm to solve the CPP problem in a grid-based environment is the wavefront algorithm (Zelinsky et al., 1993). When given a start and a goal position (which can be the same), this algorithm propagates a *wave* from the goal to the neighboring grid cells (i.e., with a breadth-first search through the state-space). When the wave reaches a new grid cell, we label the cell with the next number with respect to the highest label number of the already visited neighboring cells.

After the propagation, each grid cell will be labeled with a number corresponding to the minimum number of cells (or equivalently, the minimum number of moves/actions) an agent must visit (or equivalently, the minimum number of moves/actions it must execute) to reach the goal from a given cell. The second phase of the algorithm is as follows: the agent always visits first the unvisited neighboring cell with the highest label number, breaking ties arbitrarily. One disadvantage of this strategy is the necessity to specify the ending position. In some applications, the ending position is not important, and not specifying it allows for finding shorter paths. The algorithm runs in O(n) , where *n* is the number of states, but has no guarantee of finding an optimal solution. There exists an *on-line* variant of the wavefront algorithm (Shivashankar et al., 2011) which can be used when the environment to cover is *a priori* unknown.

Each grid cell in grid-based methods is typically a square or a rectangle (as in the wavefront algorithm). Oh et al. (2004) proposed instead to consider a grid of triangular cells which allows for a higher resolution in comparison to square or rectangular cells of similar size. However, as Galceran and Carreras (2013) mention in their survey: “*Most mobile robots are not capable of making very fine movement adjustments, and hence there is no need for ultra high resolution in coverage path planning. Therefore, the extra effort devoted to implementing a triangular grid seems not to be worthwhile*”.

Grid-based CPP methods have been proposed to cover irregularly shaped areas using UAVs (Unmanned Aerial Vehicles). For example, the algorithm of Cabreira et al., 2019b searches for a path that covers in the most “energy-efficient” way the area to be explored. In contrast, most other CPP methods for UAVs consider the number of “turning maneuvers”, thus minimizing the energy consumption only indirectly (see, e.g., Valente et al. (2013)). The algorithm of Cabreira et al. is similar to the baseline algorithm we present in Section 3.1 (the authors seem to use a variant of the ID-DFS algorithm, even though it is not clearly mentioned in their paper). They propose two pruning techniques to reduce the computation time. The former technique keeps in memory the best (i.e., smallest) cost of the solutions found to date, and prunes the current subtree whenever the length of the best current solution exceeds that cost. The latter technique reduces the computation by keeping intermediate cost in memory and computing the cost to reach newly encountered cells using the stored cost of its neighbors instead of computing the cost of travel from the initial cell. These pruning techniques cannot be applied to solve the grid-based CPP problem described in the following sections, because we consider the number of visited cells (including multiple visits) instead of the energy or cost of the path, and ID-DFS already prevents such implausible states from being visited.

Another algorithm based on a grid representation of the problem uses minimum spanning trees (Gabriely and Rimon, 2001). It can be used either as an on-line or off-line planner. The algorithm of Gabriely and Rimon, named *Spanning Tree Covering*, solves a relaxed CPP problem assuming that the environment can be discretized using squares of size twice as large as the agent. In this simplified problem, the algorithm finds an optimal solution and has a time complexity of O(n) .

When a CPP algorithm is applied to a road network (e.g., a street cleaning vehicle that needs to pass by every street), a graph representation (instead of a grid one) is more advantageous. It allows one to model environmental constraints, such as one-way roads, and *a priori* incomplete information. Many algorithms have been proposed to solve the discrete CPP using graph-based representations (see the survey by Xu (2011)). Some authors have considered the discrete CPP problem with a different objective function to be optimized. For example, instead of minimizing the number of steps required to cover a region, one might be interested in minimizing the number of rotations of the agent since, depending on the type of agent, a rotation can be costly in energy (e.g., for skid-steered robots) or in time (e.g., for differential drive robots). An algorithm based on the A* algorithm has been proposed to solve this problem (Dogru and Marques, 2018). Finally, a neural-network representation of the problem has also been proposed (Yang and Luo, 2004). One advantage of this representation is that it can handle dynamic environments.

When the environment to be covered is continuous, *cellular decomposition methods* can be used (Choset and Pignon, 1998). These methods consist in partitioning complex regions in many simpler, non-overlapping regions, called cells. These simpler regions do not contain obstacles, and are thus easy to cover. Within this approach, the CPP problem can be viewed as two different sub-problems: (1) to find a “good” cell decomposition of the environment, and (2) to find the optimal order of visits of the cells. The simplest and most popular algorithm that uses this strategy is the *boustrophedon decomposition* algorithm (Choset and Pignon, 1998). A more general type of decomposition is the *Morse decomposition* which, unlike the boustrophedon decomposition, can handle non-polygonal obstacles (Acar et al., 2002). Instead of using cellular decomposition, continuous regions can also be discretized, which allows the usage of any of the aforementioned discrete planners for the complete coverage of the environment.

For a more detailed description of traditional CPP algorithms, we refer the reader to the seminal surveys of Choset (2001) and Galceran and Carreras (2013). More recent surveys, focusing on specific variants or applications of CPP also exist. For example, in their survey, Khan et al. (2017) focus on optimized backtracking and smoothness techniques, which have not been covered previously. Finally, the review of Cabreira et al., 2019a concentrates on CPP techniques applied to unmanned aerial vehicles (UAVs).

Several works in the field are dedicated to practical applications of the CPP problem. For instance, Nasirian et al. (2021) have proposed a new representation of the problem, using a Markov Decision Process (MDP), and an algorithm based on deep reinforcement learning for finding a continuous path for a disinfectant robot that minimizes the disinfection task completion time in a hospital, to lower COVID-19 or other virus transmission risks.

All of the above algorithms have a relatively fast running time, but they either focus on a relaxed instance of the CPP problem (e.g., the aforementioned spanning-tree covering algorithm) or have no guarantee of optimality (e.g., the aforementioned wave-front algorithm). It is important to mention that there exists a reduction from the Traveling Salesman Problem (TSP) to the general discrete CPP problem, making CPP a part of the NP-complete class of problems (Mitchell, 2017). Therefore, the CPP planners described in the literature either provide approximate problem solutions, or work only within a relaxed version of the CPP problem with additional constraints on the environment. To the best of our knowledge, no discrete CPP planner that are both *optimal and general* has been proposed in the literature (other than the trivial, naive algorithm consisting on exploring the entire state-space).

The rest of the article is organized as follows. In Section 2, we formally introduce the CPP variant of interest. Section 3 and Section 4 describe, respectively, our method and the results obtained. Finally, Section 5 presents the main findings of our study, and discusses some ideas for future investigation.

## 2 Problem modeling

In this section, we mathematically define the coverage path planning theoretical framework we used in this research. Definitions 1–3 describe more formally the environment to cover, the agent and the state-space model considered in our study.

Definition 1 A 2D *environment* is an *m* × *n* grid represented by a matrix $G={\left(g_{ij}\right)}_{m\times n}$ , where *g*_*ij*_ ∈ {*O*, *X*}, and:

• *O* indicates that a cell is accessible and needs to be covered;
• *X* means that the cell is inaccessible (blocked by an obstacle).

Definition 2 An *agent* is an entity with a position somewhere on the grid. It can move to neighboring grid cells by using an action *a* from the set of actions A={(−1,0),(+1,0),(0,−1),(0,+1)} . The effect of each action is as follows.

If *p* = (*i*, *j*) denotes the agent’s current position (i.e., the agent is on the grid cell *g*_*ij*_) and it executes action *a* = (*a*_1_, *a*_2_), then its new position $\tilde{p}$ is:

$$\tilde{p}=\left\{\begin{array}{cc} \left(i+a_{1},j+a_{2}\right)\; & \;\text{if}\;\;g_{i+a_{1},j+a_{2}}=O\\ p\; & \;\text{if}\;g_{i+a_{1},j+a_{2}}=X. \end{array}\right.$$

Definition 3 A *state* is a tuple *s* = (*i*_*s*_, *j*_*s*_, *R*), where:

• (*i*_*s*_, *j*_*s*_) is the position (row, column) of the agent;
• *R* = {(*i*, *j*)∣*g*_*ij*_ = *O* and position (*i*, *j*) has not yet been explored}.

**FIGURE 2:**
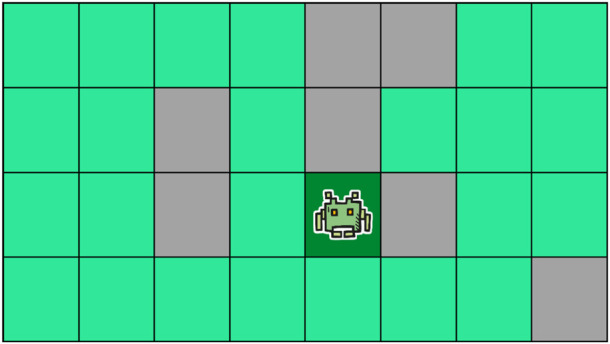
A CPP instance. The dark green, light green and gray cells represent, respectively, the initial cell, the cells that remain to be covered and the inaccessible cells.

Assuming a square grid of size *n* × *n*, the state-space (the set of all states) has cardinality $n^{2}\times 2^{n^{2}}$ . It is thus important to note that the state-space is exponentially larger than the size of the grid environment. We now formally define what we mean by a CPP problem instance, a solution to such an instance, and our optimization criterion (see Definitions 4–6), and give an example of a problem instance in Figure 2.

Definition 4 An *instance* of our CPP problem variant is given by a tuple (*G*, *s*_0_), where *G* is an environment, as defined in definition 1, and *s*_0_ = (*i*_0_, *j*_0_, *R*_0_) is the initial state, where *R*_0_ = {(*i*, *j*)∣*g*_*ij*_ = *O*}.

Definition 5 A *solution* to such an instance (*G*, *s*_0_) is an ordered list of actions *p* = ⟨*a*_1_, *a*_2_, *…* , *a*_*k*_⟩ (also called a plan) that moves the agent through positions:

$$L=\left\langle \left(i_{0},j_{0}\right),\left(i_{1},j_{1}\right),\ldots ,\left(i_{k},j_{k}\right)\right\rangle ,$$

with *R*_0_ ⊆ *L* (i.e., the final state is (*i*_*k*_, *j*_*k*_, ∅)).

Definition 6 Let P be the set of solutions (plans) of a CPP problem instance. The *objective* is to find an optimal solution $p^{⋆}={\mathrm{argmin}}_{p\in P}|p|$ . Namely, *p* ^⋆^ is a minimal ordered list of actions that solves the problem.

## 3 Proposed methods

**FIGURE 3:**
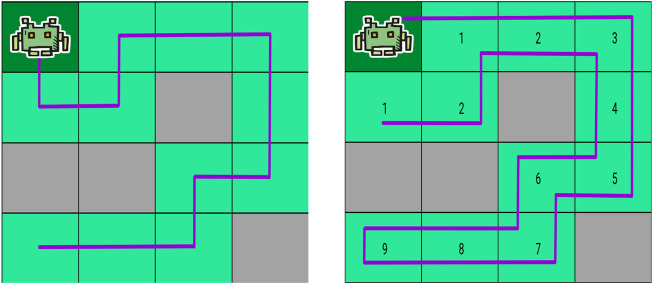
Difference in the path obtained by an optimal solver (left) and by the wavefront algorithm (right). For the wavefront algorithm, the wave values are shown inside each grid cells.

As previously noted, existing methods used to solve the grid-based CPP problem are either non-general (they work only in some specific cases) or non-optimal (they don’t necessarily provide the optimal, i.e., shortest-path, solution). To illustrate how suboptimal they can be, we present in Figure 3 the path obtained on the same 4 × 4 grid by an optimal solver (on the left) and by the wavefront algorithm described in Section 1 (on the right). The optimal solution has a length of 11, while the solution found by the wavefront algorithm has a length of 19. The optimal solution thus requires 8 moves less, which is about 40% shorter. Note that this example is a worst-case scenario for the wavefront algorithm in a 4 × 4 grid. Thus, the difference in solution quality between classical algorithms like the wavefront algorithm and optimal algorithms can be significant.

In the first part of this section, we present an *optimal* CPP planner based on the ID-DFS algorithm. Because the problem is NP-complete, the algorithm has a worst-case exponential time complexity. The second part of this section describes the two improvements we propose (i.e., loop detection and an admissible heuristic function) which preserve the optimality of the obtained solutions, while running orders of magnitude faster than an exhaustive search algorithm.

### 3.1 Iterative deepening depth-first search (ID-DFS)

Since there is no *optimal and general* discrete CPP planner described in the literature, we begin by presenting an exhaustive search planner, on which our two proposed improvements are based on.

First, we observe that our problem can be viewed as a search in a graph where every node represents a state in the state-space (as defined in Definition 3). Thus, we can theoretically use any standard graph search algorithm, such as the well-known depth-first search (DFS) and breadth-first search (BFS) algorithms. However, because of the huge size of the graph representing the state-space (i.e., the state-space is exponentially larger than the problem grid), BFS is impractical. Indeed, this search algorithm needs in the worst-case scenario to store the complete state-space in the working memory, which is too large even for a small problem grid (e.g., a 20 × 20 grid has a state-space of 200 × 2^200^ states). In practice, we can’t use BFS or other algorithms based on BFS, such as the Dijkstra and A^⋆^ algorithms.

Algorithm 1 CPP planner based on ID-DFS.
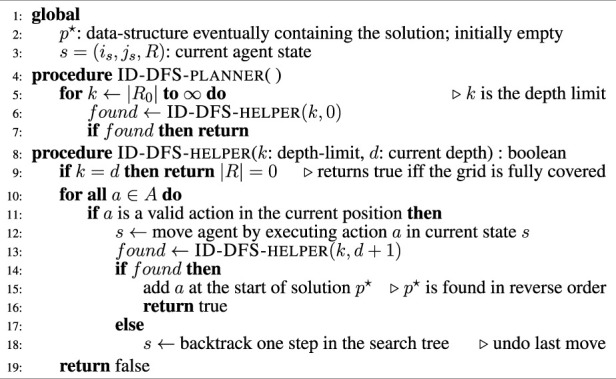

On the other hand, the DFS algorithm can go arbitrarily deep in the search tree even when the solution is close to the root (e.g., it can get stuck by expanding nodes over and over indefinitely, never backtracking and thus never finding a solution). A safeguard is to use a variant of DFS, called iterative deepening depth-first search (ID-DFS) (Russell and Norvig, 2009), which works similarly to DFS but considers a depth limit. When a solution is not found within the current depth limit *k*, the algorithm restarts its search but with the depth limit *k*+1 and continues until a solution is found. It ensures that the algorithm never goes deeper than necessary and that the algorithm terminates (if a solution exists). Algorithm 1 presents the details of a CPP planner based on ID-DFS. We recall from the previous section that *R*_0_ is the set of grid cells that need to be covered. The rest of the pseudocode should be self-explanatory.

### 3.2 Pruning using loop detection

Algorithm 1 provides an optimal solution for each problem instance and is guaranteed to terminate if a solution exists. However, it has to analyze many branches of the search tree that are not promising (i.e., that have a little chance or no chance at all to lead to an optimal solution). Our branch-and-bound planner aims at alleviating this problem by pruning the unpromising parts of the search tree during the search. There are many different types of unpromising subtrees. One such type occurs when the agent arrives in an already visited grid cell (*i*, *j*) without having covered any new grid cell since its last visit (i.e., we found a *loop* in the state-space). In order to find such loops, we introduce the matrix $M={\left(m_{ij}\right)}_{m\times n}$ , where *m*_*ij*_ is the number of grid cells that remained to be covered the last time the agent was in position (*i*, *j*). We modify Algorithm 1 to consider and update this new matrix *M*. When a new recursive call starts, and the agent is in position (*i*, *j*), a condition is inserted to check if *m*_*ij*_ ≤ |*R*|. If this condition is true, then the current path is clearly suboptimal, and the current subtree is thus pruned from the search tree.

### 3.3 Pruning using an admissible CPP heuristic function

A second way to improve Algorithm 1 is to introduce an *admissible heuristic* cost function h:S→N , i.e., a function that takes as input states *s* = (*i*, *j*, *R*) from the set of states S and returns as output a lower bound *h*(*s*) on the number of actions needed to cover the remaining uncovered grid cells (the cells in *R*). Such a heuristic can be used in two ways: (1) It allows pruning even more unpromising subtrees than what is possible using the Loop-Detection method (see Section 3.2), and (2) it allows ranking the successors of a state depending on how much promising they are, and thus finding an optimal solution faster by exploring the most promising subtrees first.

**FIGURE 4:**
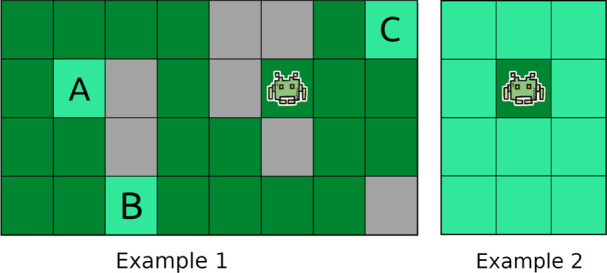
Examples explaining how the proposed heuristic works in practice.

We describe our novel heuristic and explain how it computes the lower bound using an example presented in Figure 4 (Example 1, on the left). In this figure, three grid cells (A, B and C) remain to be covered. Our heuristic computes the minimum number of actions (for each of the four possible moves) in A that need to be executed to cover the remaining cells. For example, the action corresponding to “go left” must be executed at least max(4, 3, 0) = 4 times and the action corresponding to “go right” must be executed at least max(0, 0, 2) = 2 times. To obtain a tighter lower-bound, we observe that if the agent goes 2 cells to the right, the minimum number of moves to the left will now be two more than for the previous minimum of 4, i.e., 6. We can thus add min(*left*, *right*) to the number of “left moves” and “right moves”. The same computation is carried out for the “go up” and “go down” moves. We thus finally obtain a heuristic value of:

$$4+2+\min\left(4,2\right)+1+2+\min\left(1,2\right)=12.$$

In Example 1, the obtained heuristic value of 12 is not higher than the actual number of actions necessary to cover A, B and C, and is a much more informative heuristic value than the number of uncovered cells (i.e., 3). However, under some (rare) circumstances, the number of uncovered cells can be more informative than the heuristic value computed as explained above. For instance, if we look at Example 2 in Figure 4, the heuristic value we would obtain is:

$$1+1+\min\left(1,1\right)+1+2+\min\left(1,2\right)=7,$$

which is lower than the number of uncovered cells (i.e., 11). To further improve the informativeness of our heuristic function (i.e., obtain tighter lower bounds on the remaining number of actions), our heuristic function returns the maximum of the two values.

Algorithm 2 shows more precisely how the heuristic value *h*(*s*) of a given state *s* is computed.

Algorithm 2 Heuristic cost computation.
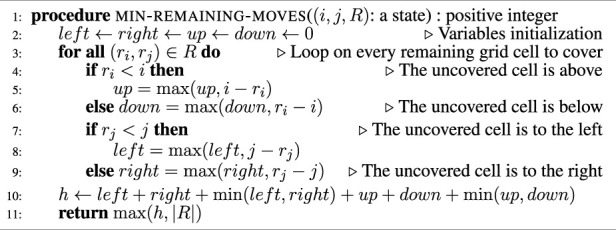

### 3.4 ID-DFS CPP planner using the proposed pruning techniques

Algorithm 3 presents the planner we obtain when modifying the ID-DFS planner (Algorithm 1) to prune the state-space using the two proposed techniques (pruning using loop detection, and pruning using the admissible heuristic function). In Algorithm 3, the added instructions related to the loop detection are colored in green, while those related to the use of the heuristic function are colored in blue.

Pruning using loop-detection is done exactly as described in Section 3.2. The matrix defined on Line four is used on Line 13 to prune the detected loops in the state-space.

The heuristic function (i.e., the function defined in Algorithm 2) is used in two ways. Firstly, it is used on Line six to provide an initial depth limit for ID-DFS. This allows the algorithm to start with a higher initial depth limit than in Algorithm 1 in the case when the heuristic value is higher than the number |*R*_0_| of cells to be covered in the initial grid. Secondly, it is used on Line 11 to prune the currently explored subtree in the state-space if *k*−*d*, i.e., the number of remaining moves that can be done before reaching the depth limit, is less than the lower-bound, given by the heuristic function, of the number of necessary moves to complete the coverage.

Algorithm 3 Heuristic cost computation.
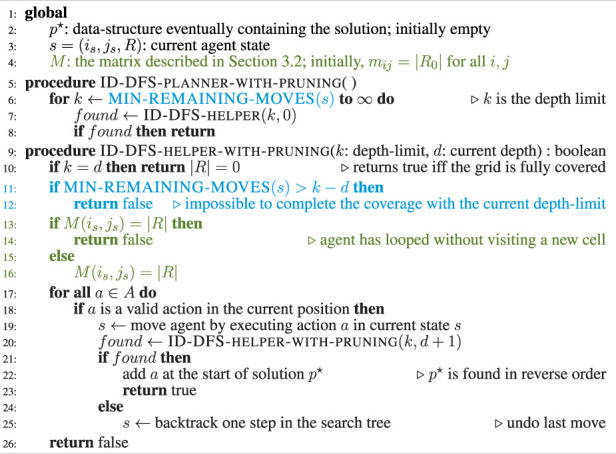

## 4 Results and analysis

The algorithms described in Section 3 were implemented in C++. The tests were carried out on a PC computer equipped with an Intel Core i5 7600k processor and 32 GB of RAM. Thanks to ID-DFS’s low memory consumption, all tested algorithms have never used more than 10 MB of RAM memory, even on the largest test environments. Thus, the memory usage is not an interesting benchmarking metric here. The obtained solutions’ quality is also not an interesting metric to compare the proposed algorithms (the baseline, ID-DFS, and the two proposed improvements, Loop-Detection and Heuristic function) because all of them always provide *optimal* solutions, i.e., plans with the same minimal length (number of actions). However, for each tested grid, our program returns the length of an optimal solution as well as the solution found by the classical (non-optimal) wavefront algorithm (described in Section 1) to give an idea of the difference in the quality of solution we can expect between an optimal and a classical, approximate algorithm.

We propose six types of synthetic grids which cover many completely different scenarios. We believe that by using these synthetic grids in our evaluation, we are able to measure more generally the strenghts and weaknesses of each proposed algorithm (compared to testing it on a specific real-world grid-environment, which would have a particular shape and not cover as many scenarios).

**FIGURE 5:**
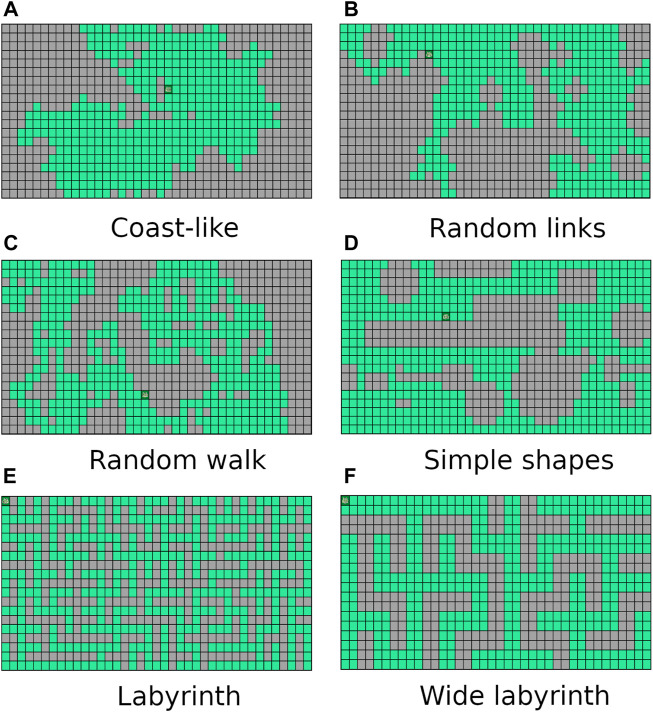
The six types of generated grids in our benchmark: **(A)** Coast-like, **(B)** Random links, **(C)** Random walk, **(D)** Simple shapes, **(E)** Labyrinth, **(F)** Wide-labyrinth.

Every planner was tested with each of the six kinds of artificial grids shown in Figure 5. Type (a) grids were generated with the Diamond-Square algorithm. They have the shape of a coast (Fournier et al., 1982). Type (b) grids include cells with randomly added “links” between neighboring positions on the grid. Type (c) grids mimic a random walk on a grid. Type (d) grids were generated by randomly placing simple shapes (triangles, discs and rectangles). Type (e) grids are perfect labyrinths (i.e., labyrinths with no cycle). Finally, type (f) are wide labyrinths (i.e., labyrinths were each "path" has a width of 2 cells). All grid types were generated with an inaccessible cells density of (50 ± 1)%. To measure the performance of our algorithms, we ran each of them 50 times for each parameter combination (type of grid, size of grid) using randomly generated grids, and taking the average of the obtained results.

**FIGURE 6:**
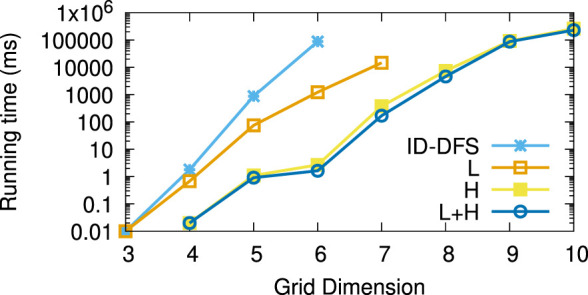
Average running times (in ms) for the type (a) (coast-like) grids.

**FIGURE 7:**
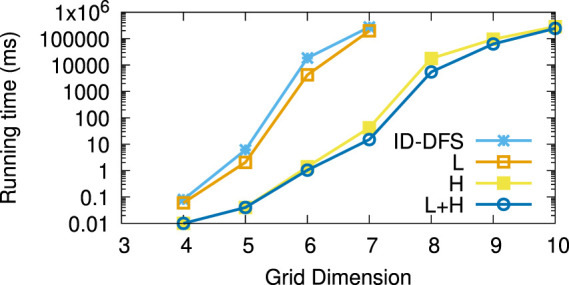
Average running times (in ms) for the type (b) (random links) grids.

**FIGURE 8:**
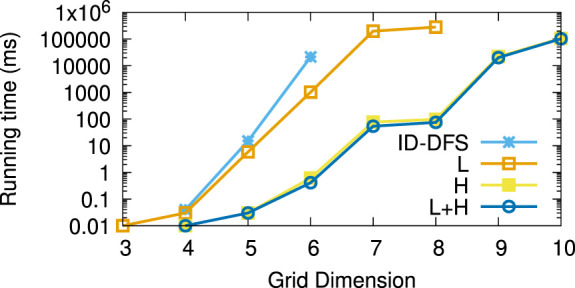
Average running times (in ms) for the type (c) (random walk) grids.

**FIGURE 9:**
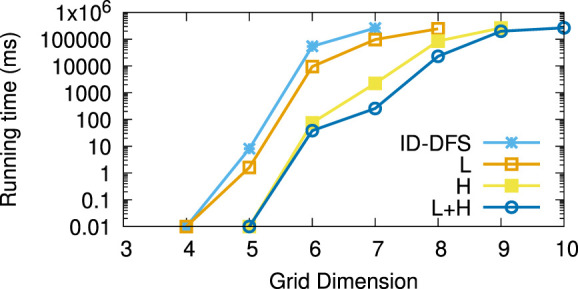
Average running times (in ms) for the type (d) (simple shapes) grids.

**FIGURE 10:**
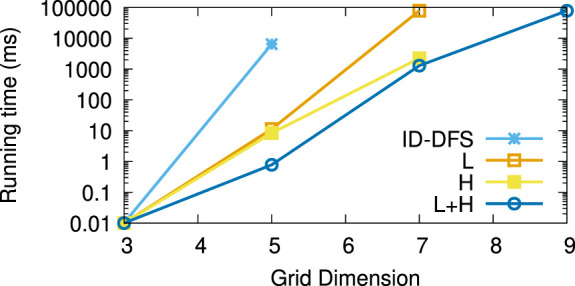
Average running times (in ms) for the type (e) (labyrinth) grids.

**FIGURE 11:**
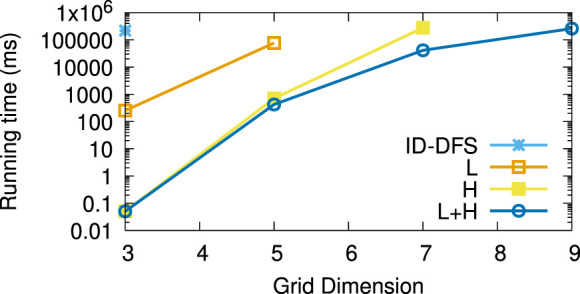
Average running times (in ms) for the type (f) (wide labyrinth) grids.

Table 1 reports the average running times measured for each planner on each instance of the considered types of test grids. Every grid we generated had the same number of rows and columns, shown in column *Size*. The columns L and H, respectively, stand for our two improvements over the ID-DFS (Algorithm 1) planner, i.e., (L)oop detection and (H)euristic pruning. In Table 1, the character ’-’ means that the planner failed to solve the problem within 5 min. The length of an optimal solution (which is also the length of the solution obtained by each of our proposed planners, since all of them are optimal) is given by column OS. For reference, we also provide the length of the solution obtained by the classical wavefront algorithm. Figures 6–11 illustrate the obtained results graphically.

**TABLE 1:** Average running times (in ms) obtained by the proposed planners. Columns “OS” and “WFS” give the length of an optimal solution and of the solution obtained with the approximate wavefront solver. The symbol ‘-’ indicates when a solver could not solve an instance within 5 min.

Type	Size	ID-DFS	L	H	L + H	OS	WFS
(a)	3	0.01	0.01	0.00	0.00	4	4
(a)	4	1.79	0.68	0.02	0.02	9	9
(a)	5	877.45	75.35	1.10	0.91	14	16
(a)	6	87,595.50	1220.18	2.68	1.63	18	19
(a)	7	-	14,598.20	377.89	169.61	24	27
(a)	8	-	-	7369.90	4605.85	34	38
(a)	9	-	-	91,806.70	86,891.90	42	47
(a)	10	-	-	259,001.00	228,610.00	48	52
(b)	3	0.00	0.00	0.00	0.00	3	3
(b)	4	0.08	0.06	0.01	0.01	8	9
(b)	5	6.10	2.05	0.04	0.04	12	13
(b)	6	18,475.70	4225.29	1.40	1.04	19	20
(b)	7	275,006.00	196,072.00	41.98	14.91	24	26
(b)	8	-	-	17,688.50	5481.63	33	35
(b)	9	-	-	94,546.60	63,041.10	40	43
(b)	10	-	-	285,868.00	244,529.00	47	52
(c)	3	0.00	0.01	0.00	0.00	5	5
(c)	4	0.04	0.03	0.01	0.01	7	9
(c)	5	15.03	5.84	0.03	0.03	13	14
(c)	6	21,457.50	1024.13	0.62	0.41	18	21
(c)	7	-	197,872.00	76.95	53.62	26	27
(c)	8	-	280,669.00	97.45	75.26	32	36
(c)	9	-	-	22,104.50	20,105.50	39	42
(c)	10	-	-	105,775.00	102,159.00	52	57
(d)	3	0.00	0.00	0.00	0.00	2	2
(d)	4	0.01	0.01	0.00	0.00	6	6
(d)	5	8.03	1.61	0.01	0.01	10	12
(d)	6	54,370.60	9442.40	74.96	38.26	22	25
(d)	7	260,308.00	96,891.80	2219.24	257.49	27	32
(d)	8	-	243,374.00	85,053.10	22,908.10	37	42
(d)	9	-	-	260,703.00	197,377.00	39	52
(d)	10	-	-	-	266,157.00	57	62
(e)	3	0.01	0.01	0.01	0.01	7	7
(e)	5	6443.80	11.29	8.78	0.38	22	26
(e)	7	-	78,432.00	2241.60	1291.27	45	53
(e)	9	-	-	-	78,432.14	73	83
(f)	3	218,413.00	251.60	0.05	0.04	26	27
(f)	5	-	76,982.30	702.31	423.79	67	67
(f)	7	-	-	278,412.00	41,247.00	109	124
(f)	9	-	-	-	259,001.00	157	181

As we can see, the L variant is about an order of magnitude faster than the baseline ID-DFS implementation. The H variant is many orders of magnitude faster than the baseline ID-DFS. The combination of both improvements (L + H) led to an even greater speedup, albeit the difference between H and L + H is smaller than the difference between ID-DFS and H, or between ID-DFS and L. This is not surprising, since there is an overlap between the unpromising states detected due to the loop detection and those detected by using the proposed heuristic function. The relative performance of the tested algorithms is the same in every type of test grids (ID-DFS ≺ L ≺ H ≺ L + H). That being said, since the overhead of L + H over H (in terms of the memory usage for the matrix designed to detect loops, and in terms of an added programming complexity) is minimal, there is no reason not to always use L + H in practice.

Table 2 reports the average speedup factors provided by each of the three proposed algorithmic improvements (L, H, L + H) on the six types of grids. It also reports (in the ASLD column) the Average Solution Length Difference between an optimal solution (returned by our algorithms) and an approximate solution (returned by the wavefront algorithm). As we can see, L + H has been able to find optimal solutions thousands to millions of times (depending on the grid type) faster than our baseline ID-DFS planner. Furthermore, we see that the average solution length difference between an optimal and approximate solution is at 12% (over all grid types), which can be an important improvement in some real-world problems.

**TABLE 2:** Average speedup factors produced by each of the three proposed algorithmic improvements on the six types of grids.

Type	L	H	L + H	ASLD
(a) Coast-Like	68.26	23,282.83	34,560.45	1.10
(b) Random Links	1.47	6757.72	18,342.99	1.08
(c) Random Walk	20.85	32,534.20	47,716.82	1.10
(d) Simple Shapes	2.96	137.17	1063.99	1.17
(e) Labyrinth	570.25	768.03	8156.72	1.15
(f) Labyrinth-Wide	868.10	4,368260	5,460325	1.11
Average	255.31	738,623.32	928,361.00	1.12

## 5 Conclusion

This paper considers the highly relevant optimal complete coverage path planning (CPP) problem. We first briefly reviewed practical applications of CPP. Then, we showed how an exhaustive algorithm based on iterative deepening depth-first search (ID-DFS) can be efficiently accelerated using a branch-and-bound approach. The proposed improvements (loop-detection and an admissible heuristic function) allow the planner to find an optimal CPP solution many orders of magnitude faster than a baseline ID-DFS implementation, making it highly suitable for practical applications where having an optimal solution is important.

As future work, we plan to develop and test a method similar to particle swarm optimization (PSO) considering an initial particle that splits the problem into several sub-problems every time there is more than one eligible neighbor. The splitting process will take place until the number of particles reaches a certain threshold. When this happens, a pruning process destroying the least promising particles can be carried out according to an evaluation heuristic to be determined. We also envisage using clustering algorithms (Mirkin, 2005) to decompose a given grid into smaller, mostly independent sub-grids (i.e., similar to cellular decomposition, but for grid environments), which could be covered optimally one by one. Such an algorithm could also be easily parallelized.

## Data Availability

The raw data supporting the conclusions of this article will be made available by the authors, without undue reservation.
